# ﻿Two new species and one new combination of *Ophiocordyceps* (Hypocreales, Ophiocordycipitaceae) in Guizhou

**DOI:** 10.3897/mycokeys.102.113351

**Published:** 2024-02-29

**Authors:** Xing-Can Peng, Ting-Chi Wen, De-Ping Wei, Yu-Hong Liao, Yi Wang, Xian Zhang, Gui-Ying Wang, Yun Zhou, Khanobporn Tangtrakulwanich, Jian-Dong Liang

**Affiliations:** 1 Basic Medical School, Guizhou University of Traditional Chinese Medicine, Guiyang 550002, China; 2 National Key Laboratory of Green Pesticide, Key Laboratory of Green Pesticide and Agricultural Bioengineering, Ministry of Education, Guizhou University, Guiyang 550025, Guizhou, China; 3 Engineering Research Center of Southwest Bio-Pharmaceutical Resources, Ministry of Education, Guizhou University, Guiyang 550025, Guizhou, China; 4 Center of Excellence in Fungal Research, Mae Fah Luang University, Chiang Rai 57100, Thailand; 5 School of Science, Mae Fah Luang University, Chiang Rai 57100, Thailand; 6 Guizhou Key Laboratory of Edible Fungi Breeding, Guiyang 550006, China; 7 School of Pharmacy, Guizhou University, Guiyang 550025, Guizhou, China; 8 Shenzhen Ainiang Biotechnology Co., Ltd., Shenzhen 518118, China; 9 Shenzhen Longgang Buji Middle School, Shenzhen 518112, China

**Keywords:** Entomopathogenic fungi>, morphology, phylogenetic, two new taxa

## Abstract

*Ophiocordyceps* is the largest genus in Ophiocordycipitaceae and has a broad distribution with high diversity in subtropical and tropical regions. In this study, two new species, pathogenic on lepidopteran larvae are introduced, based on morphological observation and molecular phylogeny. *Ophiocordycepsfenggangensis***sp. nov.** is characterised by having fibrous, stalked stroma with a sterile tip, immersed perithecia, cylindrical asci and filiform ascospores disarticulating into secondary spores. *Ophiocordycepsliangii***sp. nov.** has the characteristics of fibrous, brown, stipitate, filiform stroma, superficial perithecia, cylindrical asci and cylindrical-filiform, non-disarticulating ascospores. A new combination *Ophiocordycepsmusicaudata* (syn. *Cordycepsmusicaudata*) is established employing molecular analysis and morphological characteristics. *Ophiocordycepsmusicaudata* is characterised by wiry, stipitate, solitary, paired to multiple stromata, yellowish, branched fertile part, brown stipe, immersed perithecia, cylindrical asci and cylindrical-filiform, non-disarticulating ascospores.

## ﻿Introduction

Hypocreales is a fungal order enriched in arthropod-pathogens, which are taxonomically placed in Clavicipitaceae, Cordycipitaceae, Ophiocordycipitaceae and Polycephalomycetaceae (Luangsa-Ard et al. 2018; Wei et al. 2021a; Xiao et al. 2023). Entomopathogens in these four families can infect many orders of insects and arachnids (Luangsa-Ard et al. 2018; Wei et al. 2022). Hypocrealean entomopathogens can infect various developmental stages of the insect, from larva, pupa to nymph and adults (Luangsa-Ard et al. 2018). For example, *Ophiocordycepsacicularis* is a parasite on larvae of Coleoptera (Sung et al. 2007), *Cordycepsmorakotii* on pupa of Hymenoptera (Tasanathai et al. 2016), *Cordycepscocoonihabita* on cocoons of Lepidoptera (Wang et al. 2020), *Ophiocordycepsasiana* on adults of Hemiptera (Khao-ngam et al. 2021), *Ophiocordycepsdipterigena* on adults of Diptera (Quandt et al. 2014) and *Simplicilliumyunnanense* on the Araneae (Wang et al. 2020).

Ophiocordycipitaceae contains more than 500 species and about three out of five species are distributed in its type genus *Ophiocordyceps*. *Ophiocordyceps* was established by Petch (1931) to accommodate four species with non-disarticulating ascospores, as well as clavate asci with thickened apices: *Ophiocordycepsblattae*, *O.unilateralis*, *O.rhizoidea* and *O.peltata*. Subsequently, an increasing number of species were transferred from *Cordyceps* into *Ophiocordyceps* (Sung et al. 2007; Quandt et al. 2014). *Ophiocordyceps* are characterised by dark, fibrous, wiry, pliant stromata, superficial to completely immersed perithecia, cylindrical asci with thickened cap, fusiform to filiform ascospores disarticulating or non-disarticulating (Sung et al. 2007; Xiao et al. 2019). The asexual genera associated with *Ophiocordyceps* are *Hirsutella*, *Hymenostilbe*, *Paraisaria*, *Stilbella* and *Syngliocladium* (Sung et al. 2007; Quandt et al. 2014; Yang et al. 2021). By employing multigene phylogeny, Quandt et al. (2014) updated the generic composition of Ophiocordycipitaceae and accepted *Drechmeria*, *Harposporium*, *Ophiocordyceps*, *Polycephalomyces*, *Purpureocillium* and *Tolypocladium* in this family; meanwhile, twelve genera including *Cordycepioideus*, *Didymobotryopsis*, *Didymobotrys*, *Hirsutella*, *Hymenostilbe*, *Mahevia*, *Paraisaria*, *Sorosporella*, *Syngliocladium*, *Synnematium*, *Trichosterigma* and *Troglobiomyces* were rejected in favour of *Ophiocordyceps*, following the principle of “one fungus one name”. Mongkolsamrit et al. (2019) resurrected the genus *Paraisaria* in Ophiocordycipitaceae. Crous et al. (2020) and Araújo et al. (2022) established *Hantamomyces* and *Torrubiellomyces*, respectively. Xiao et al. (2023) transferred *Polycephalomyces*, *Perennicordyceps* and *Pleurocordyceps* from Ophiocordycipitaceae to a new family, Polycephalomycetaceae, based on biphasic analyses. Therefore, Ophiocordycipitaceae currently contains eight genera, namely *Drechmeria*, *Hantamomyces*, *Harposporium*, *Ophiocordyceps*, *Paraisaria*, *Purpureocillium*, *Tolypocladium* and *Torrubiellomyces*.

In this study, we collected three entomoapthogenic fungi> from lepidoptera larvae found in disturbed forests in Guizhou Province, China. These specimens have typical characters of *Ophiocordyceps* in terms of the macro- and micro-morphologies. This study attempts to reveal their taxonomic placements, based on morphological characteristics and molecular analysis of the combined LSU, ITS, SSU, *tef1-α*, *rpb1* and *rpb2* dataset.

## ﻿Materials and methods

### ﻿Collection, isolation and morphological study

Specimens were scanned from the ground of disturbed forests in Guizhou Province, China. Three species were found to infect lepidopteran larvae and their stromata protruded from the ground. Amongst them, the hosts of specimen HKAS 125848 were found completely immersed into soil. The host of specimen GACP SY22072880 was found semi-immersed into soil. The specimen HKAS 125845 was found on leaf litter. Macro-morphological characteristics of fresh collections were documented with a camera (Canon 6D) and locations were recorded with Biotracks in the field. The specimens were collected into a plastic box and transported to the laboratory for subsequent studies. The culture of the specimens was obtained by transferring a small mass of mycelium inside the body of the host into potato dextrose agar (PDA) using a sterile inoculation needle and incubated at 25 °C (Wei et al. 2021b). A Leica stereomicroscope (Leica S9E) was used to examine and section the fruiting bodies. Sections of fertile head were mounted on glass slides with a drop of ultrapure water and covered with a cover slip. A Leica compound microscope (Leica DM2500) was used to photograph and measure perithecia, asci, peridium, apical cap, ascospores and secondary ascospores. The fruiting bodies were dried with allochroic silica gel and deposited in the Herbarium of Cryptogams, Kunming Institute of Botany of the Chinese Academy of Sciences (**KUN-HKAS**), the cultures being deposited in the Herbarium of Guizhou University (**GACP**).

### ﻿DNA extraction, PCR amplification and sequencing

DNA was extracted from mycelium inside the body of insect hosts and from fresh mycelium on PDA medium using DNA extraction kit (Fungal gDNA Isolation Kit, Biomiga, CA, USA), following the protocol of the manufacturer. The obtained total genomic DNA was stored at -20 °C. Six loci including the partial large subunit rRNA gene (LSU), internal transcribed spacer including the 5.8S rDNA gene (ITS), the partial small subunit rRNA gene (SSU), the translation elongation factor 1-alpha gene (*tef1-α*), the partial RNA polymerase II largest subunit (rpb1) and the partial RNA polymerase II second largest subunit (*rpb2*) were amplified and sequenced. The primers LROR/LR5 were used for LSU (Vilgalys and Hester 1990), ITS5/ITS4 for ITS (White et al. 1990), NS1/NS4 for SSU (White et al. 1990), EF1-983F/EF1-2218R for *tef1-α* (Rehner and Buckley 2005), CRPB1A/RPB1Cr for *rpb1* (Castlebury et al. 2004) and fRPB2-5f/fRPB2-7cR for *rpb2* (Castlebury et al. 2004). The Polymerase Chain Reaction (PCR) was performed in a 50 µl volumes consisting of 22 µl PCR mixture (2× Taq PCR StarMix with Loading Dye, GenStar) which contains *Taq* DNA polymerase, dNTPs, Mg^2+^, a reaction buffer and stabiliser, 20 µl of double distilled water, 2 µl of each primer and 4 µl of DNA template. Amplifications were carried out using a BioRAD T100 Thermal Cycler (Singapore) with the following conditions: (1) initialisation at 94 °C for 3 min, for ITS (2) 33 cycles of denaturation at 94 °C for 30 sec, annealing at 51 °C for 50 sec and extension at 72 °C for 45 sec; for SSU and LSU (2) 33 cycles of denaturation at 94 °C for 30 sec, annealing at 50 °C for 30 sec and extension at 72 °C for 2 min; for *tef1-α* (2) 33 cycles of denaturation at 94 °C for 30 sec, annealing at 58 °C for 50 sec and extension at 72 °C for 1 min; for *rpb1* and *rpb2* (2) 33 cycles of denaturation at 94 °C for 30 sec, annealing at 51 °C for 40 sec and extension at 72 °C for 1 min 20 sec and followed by (3) final extension at 72 °C for 10 min. The PCR products were sent to Tsingke Biological Technology in Chongqing, China, for sequencing using the above primers. The generated sequences were edited manually for excluding ambiguous region with BioEdit v.7.0.5.3 (Hall et al. 2011). The accession numbers and hosts are listed in Table 1.

**Table 1.** GenBank accession numbers of the taxa used in the phylogenetic analyses, the newly- generated sequences are in bold, ^T^ Represents type strain, type specimens or neotype.

Current name	Voucher	host	LSU	ITS	SSU	*tef1*-α	*rpb1*	*rpb2*	References
*Cordycepsmilitaris*	OSC 93623	Lepidoptera	AY184966	JN049825	AY184977	DQ522332	DQ522377	AY545732	Kepler et al. 2012
	YFCC 6587		MN576818		MN576762	MN576988	MN576878	MN576932	Wang et al. 2020
*Drechmeriabalanoides*	CBS 250.82^T^		AF339539		AF339588	DQ522342		DQ522442	Sung et al. 2007
*D.gunnii*	OSC 76404	Lepidoptera	AF339522	JN049822	AF339572	AY489616	AY489650	DQ522426	Luangsa-Ard et al. 2018
*D.panacis*	CBS 142798^T^	Apiales	MF588897	MF588878	MF588890	MF614144			Yeh et al. 2021
*D.zeospora*	CBS 335.80^T^		AF339540	MH861269	AF339589	EF469062	EF469091	EF469109	Vu et al. 2019
*Harposporiumanguillulae*	ARSEF 5593		AY636081						Chaverri et al. 2005
*Har.cycloides*	ARSEF 5599		AY636083						Chaverri et al. 2005
*Har.harposporiferum*	ARSEF 5472^T^		NG_060621		AF339569				Sung et al. 2001
*Har.helicoides*	ARSEF 5354	Nematode	AF339527		AF339577				Sung et al. 2001
*Hirsutellacitriformis*	ARSEF1 035	Hemiptera	KM652105	KM652153	KM652064	KM651989	KM652030		Simmons et al. 2015
	ARSEF 1446	Hemiptera	KM652106	KM652154	KM652065	KM651990	KM652031		Simmons et al. 2015
*Hir.fusiformis*	ARSEF 5474	Coleoptera	KM652110		KM652067	KM651993	KM652033		Simmons et al. 2015
*Hir.gigantea*	ARSEF 30	Hymenoptera	JX566977			JX566980	KM652034		Simmons et al. 2015
*Hir.kuankuoshuiensis*	GZUIFR-2012KKS3-1	Lepidoptera	KY415582	KY415575		KY415590	KY945360		Qu et al. 2021
*Hir.radiata*	ARSEF 1369	Diptera	KM652119		KM652076	KM652002	KM652042		Simmons et al. 2015
*Hir.shennongjiaensis*	GZUIFR-Snj121022^T^	Dermaptera	KY945357	KT390721			KY945364		Zou et al. 2016
*Ophiocordycepsacicularis*	OSC 110987	Coleoptera	EF468805		EF468950	EF468744	EF468852		Sung et al. 2007
*O.agriotidis*	ARSEF 5692	Coleoptera	DQ518754	JN049819	DQ522540	DQ522322	DQ522368	DQ522418	Kepler et al. 2012
*O.alboperitheciata*	YHH 16755^T^	Lepidoptera	MT222278			MT222279	MT222280	MT222281	Fan et al. 2021
*O.appendiculata*	NBRC 106959	Coleoptera	JN941412	JN943325	JN941729	AB968578	JN992463	AB968540	Ban et al. 2015
*O.araracuarensis*	HUA 186135	Hemiptera	KC610769	KP200891	KC610788	KC610738	KF658665	KC610716	Sanjuan et al. 2015
*O.asiatica*	BCC 86435	Blattodea	MH753676	MH754723			MK214106	MK214092	Tasanathai et al. 2019
*O.bidoupensis*	YHH 20036^T^	Coleoptera			OK571396	OK556893	OK556897	OK556899	Zou et al. 2022
*O.brunneiperitheciata*	TBRC 8100	Lepidoptera	MF614658			MF614643		MF614685	Luangsa-Ard et al. 2018
	BCC 49312	Lepidoptera	MF614660			MF614642		MF614686	Luangsa-Ard et al. 2018
*O.coccidiicola*	NBRC 100682		AB968419	AB968404	AB968391	AB968583		AB968545	Ban et al. 2015
*O.communis*	BCC 1874	Blattodea	MH753679	MH754725		MK284267	MK214109	MK214095	Tasanathai et al. 2019
*O.crinalis*	GDGM 17327	Lepidoptera	KF226254		KF226253	KF226256	KF226255		Wang et al. 2014
*O.delicatula*	ARSEF 14442^T^	Hemiptera			MZ198251	MZ246828	MZ246829		Clifton et al. 2021
*O.elongata*	OSC 110989	Lepidoptera	EF468808			EF468748	EF468856		Sung et al. 2007
*O.entomorrhiza*	KEW 53484	Lepidoptera	EF468809	JN049850	EF468954	EF468749	EF468857	EF468911	Quandt et al. 2014
***Ophiocordycepsfenggangensis***	**HKAS 125848^T^**	**Lepidoptera**	**OR527542**	**OR527535**		**OR526346**	**OR526351**		**This study**
	**GACP FG21042850**	**Lepidoptera**	**OR527541**	**OR527534**	**OR527538**	**OR526345**	**OR526350**	**OR526353**	**This study**
*O.flabellata*	YFCC 8795^T^	Hymenoptera	OL310724		OL310721	OL322688	OL322687	OL322695	Tang et al. 2023b
*O.formosana*	TNM F13893	Coleoptera			KJ878908	KJ878956	KJ878988	KJ878943	Quandt et al. 2014
*O.fusiformis*	BCC 93025^T^	Blattodea	MZ675422	MZ676743		MZ707849	MZ707855	MZ707805	Tasanathai et al. 2022
*O.gracillima*	HUA 186132	Coleoptera	KC610768	KF937353		KC610744	KF658666		Sanjuan et al. 2015
***Ophiocordycepsliangii***	**HKAS 125845^T^**	**Lepidoptera**	**OR527543**	**OR527536**	**OR527539**	**OR526347**			**This study**
	**GACP LB22071253**	**Lepidoptera**	**OR527544**	**OR527537**	**OR527540**	**OR526348**		**OR526354**	**This study**
*O.macroacicularis*	NBRC 105888	Lepidoptera	AB968417	AB968401	AB968389	AB968575		AB968537	Ban et al. 2015
*O.melolonthae*	OSC 110993	Coleoptera	DQ518762		DQ522548	DQ522331	DQ522376		Spatafora et al. 2007
*O.monacidis*	MF74	Hymenoptera	KX713605		KX713647		KX713712		Araújo et al. 2018
*O.mosingtoensis*	BCC 30904	Blattodea	MH753686	MH754732		MK284273	MK214115	MK214100	Tasanathai et al. 2019
*O.multiperitheciata*	BCC 22861	Lepidoptera	MF614656			MF614640	MF614670	MF614683	Araújo et al. 2018
***Ophiocordycepsmusicaudata***	**GACP SY22072879**	**Lepidoptera**	**OR527545**			**OR526349**	**OR526352**		**This study**
*O.naomipierceae*	DAWKSAN^T^	Hymenoptera	KX713589		KX713664		KX713701		Araújo et al. 2018
*O.nigra*	TNS 16250	Coleoptera			KJ878942	KJ878987	KJ879021		Quandt et al. 2014
*O.nigrella*	EFCC 9247	Lepidoptera	EF468818	JN049853	EF468963	EF468758	EF468866	EF468920	Sung et al. 2007
*O.nooreniae*	BRIP 55363^T^	Hymenoptera	KX673810		KX673811	KX673812		KX673809	Crous et al. 2016
*O.ovatospora*	YHH 2206001^T^	Blattodea	OP295113	OP295105	OP295110	OP313801	OP313803	OP313805	Tang et al. 2022
*O.pseudocommunis*	BCC 16757	Blattodea	MH753687	MH754733		MK284274	MK214117	MK214101	Tasanathai et al. 2019
*O.pseudorhizoidea*	BCC 86431	Blattodea	MH753674	MH754721		MK284262	MK751469	MK214090	Tasanathai et al. 2019
*O.purpureostromata*	TNS F18430	Coleoptera	KJ878897		KJ878931	KJ878977	KJ879011		Quandt et al. 2014
*O.ravenelii*	OSC 110995	Coleoptera	DQ518764		DQ522550	DQ522334	DQ522379	DQ522430	Spatafora et al. 2007
*O.robertsii*	KEW 27083	Lepidoptera	EF468826			EF468766			Sung et al. 2007
*O.sinensis*	EFCC 7287	Lepidoptera	EF468827	JN049854	EF468971	EF468767	EF468874	EF468924	Sung et al. 2007
*O.spataforae*	OSC 128575	Hemiptera	EF469079	JN049845	EF469126	EF469064	EF469093	EF469110	Sung et al. 2007
*O.unilateralis*	OSC 128574	Hymenoptera	DQ518768		DQ522554	DQ522339	DQ522385	DQ522436	Spatafora et al. 2007
*Paraisariaamazonica*	HUA 186143	Orthoptera	KJ917571		KJ917562	KM411989	KP212902	KM411982	Sanjuan et al. 2015
*Par.blattarioides*	HUA 186093	Blattodea	KJ917570		KJ917559	KM411992	KP212910		Sanjuan et al. 2015
	HUA 186108	Blattodea	KJ917569		KJ917558		KP212912	KM411984	Sanjuan et al. 2015
*Par.coenomyiae*	NBRC 108993^T^	Diptera	AB968412	AB968396	AB968384	AB968570		AB968532	Ban et al. 2015
*Par.gracilioides*	HUA 186092	Coleoptera	KJ130992		KJ917555		KP212915		Araújo et al. 2018
*Par.gracilis*	EFCC 3101	Lepidoptera	EF468810		EF468955	EF468750	EF468858	EF468913	Araújo et al. 2018
*Par.heteropoda*	EFCC 10125	Hemiptera	EF468812	JN049852	EF468957	EF468752	EF468860	EF468914	Sung et al. 2007
*Par.orthopterorum*	TBRC 9710	Orthoptera	MK332582	MH754743		MK214081	MK214085		Mongkolsamrit et al. 2019
*Par.phuwiangensis*	BBH 43491	Coleoptera	MK192058	MH188542			MH211351		Mongkolsamrit et al. 2019
*Par.tettigonia*	GZUH CS14062709^T^	Orthoptera		KT345954	KT345955	KT375440	KT375441		Wen et al. 2016
*Par.yodhathaii*	TBRC 8502	Coleoptera	MH201168	MH188540		MH211354	MH211350		Mongkolsamrit et al. 2019
*Pur.lavendulum*	FMR 10376	Soil	FR775489			FR775516	FR775512		Perdomo et al. 2013
*Pur.lilacinum*	CBS 431.87	Tylenchida	EF468844	AY624188		EF468791	EF468897	EF468940	Kepler et al. 2012
*Pur.takamizusanense*	NHJ 3582	Hemiptera	EU369034		EU369097	EU369015			Johnson et al. 2009
*Tolypocladiumbacillisporum*	C53^T^	Eurotiales	LC684523	LC684523	LC684523	LC684526			Yamamoto et al. 2022
*Tol.cylindrosporum*	ARSEF 2920^T^	Soil	MH871712	MG228381		MG228390	MG228384	MG228387	Vu et al. 2019
*Tol.inflatum*	OSC 71235	Coleoptera	EF469077	JN049844	EF469124	EF469061	EF469090	EF469108	Kepler et al. 2012
*Tol.ophioglossoides*	NBRC 106332	Eurotiales	JN941409	JN943322	JN941732		JN992466		Schoch et al. 2012
*Tol.paradoxum*	NBRC 100945		JN941410	JN943323	JN941731	AB968599	JN992465	AB968560	Ban et al. 2015
*Torrubiellomyceszombiae*	NY04434801^T^	Hypocreales	ON493602		ON493543	ON513396	ON513398	ON513402	Araújo et al. 2022
	Polyceph	Hypocreales				ON513394			Araújo et al. 2022

Abbreviations: **ARSEF**: Agricultural Research Service Entomopathogenic Fungus Collection, USDA, USA; **BBH**: BIOTEC Bangkok Herbarium, Thailand; **BCC**: BIOTEC Culture Collection, Klong Luang, Thailand; **BRIP**: Queensland Plant Pathology Herbarium, Australia; **C**: Medical Mycology Research Center, Chiba University, Japan; **CBS**: Centraalbureau voor Schimmelcultures, Utrecht, the Netherlands; **EFCC**: Entomopathogenic Fungal Culture Collection, Chuncheon, Korea; **FMR**: Culture Collection, Facultat de Medicina i Ciències de la Salut, Reus, Spain; **GDGM**: the Fungal Herbarium of Guangdong Institute of Microbiology, China; **GZUH**/**GACP**: Herbarium of Guizhou University, China; **GZUIFR**: Institute of Fungal Resources of Guizhou University, China; **HKAS**: Kunming Institute of Botany, Academia Sinica, China; **HUA**: Herbarium Antioquia University, Medellin, COL; **KEW**: mycology collection of Royal Botanical Garden, Surrey, UK; **NBRC**: Biological Resource Center, the National Institute of Technology and Evaluation, Japan; **NHJ**: Nigel Hywel-Jones personal collection, Thailand; **NY**: The New York Botanical Garden Herbarium, US; **OSC**: Oregon State University Herbarium, Corvallis, Oregon, USA; **TBRC**: Thailand Bioresources Research Center, Thailand; **YFCC**: Yunnan Fungal Culture Collection of Yunnan University, China; **YHH**: Yunnan Herbal Herbarium, China.

### ﻿Sequence alignment and phylogenetic analyses

The taxa used for phylogenetic analyses were selected, based on BLAST search results and related references (Sung et al. 2007; Quandt et al. 2014; Sanjuan et al. 2015; Araújo et al. 2018; Luangsa-Ard et al. 2018). Each locus was independently aligned with the representative sequences using MAFFT v.7 (Katoh and Standley 2013; Katoh et al. 2019). Uninformative gaps and ambiguous regions were removed using Trimal v.1.2 (Capella-Gutiérrez et al. 2009). Trimmed alignments were combined with SequenceMatrix 1.8 (Vaidya et al. 2011). The final combined dataset was deposited on TreeBASE (accession URL: http://purl.org/phylo/treebase/phylows/study/TB2:S30990) and used for Maximum Likelihood analysis and Bayesian analysis. AliView (Larsson 2014) was used to convert format with NEXUS file for Bayesian Inference analysis and FASTA file for Maximum Likelihood analysis. Two strains of *Cordycepsmilitaris* (BCC 56302 and YFCC 6587) were selected as outgroup taxa.

Maximum Likelihood (ML) analysis was performed using IQ-TREE 1.6.12 with branch support being estimated from 5000 ultrafast bootstraps (http://iqtree.cibiv.univie.ac.at/, accessed on 04 Sep 2023, Minh et al. (2020)). MrModelTest v. 2.3 (Nylander 2004) as implemented in MrMTgui v.1.0. (Nuin 2007) was used to determine the best-fit evolution model for Bayesian Inference analyses under the Akaike Information Criterion (AIC). The best-fit substitution model GTR+I+G was decided for LSU, ITS, SSU, *tef1-α* and *rpb2* and HKY+I+G for *rpb1*. MrBayes on XSEDE (3.2.7a) in the CIPRES Science Gateway was utilised to perform Bayesian analysis using Markov Chain Monte Carlo sampling (MCMC). Six simultaneous Markov chains were run for 100,000,000 generations and trees were sampled every 1000 generations. The first 20% of the trees were discarded, as they represented the burn-in phase of the analyses, while the remaining trees were used for calculating posterior probabilities (PP) in the majority rule consensus tree. Bayesian Inference trees convergence was declared when the average standard deviation reached 0.01. The trees were viewed with FigTree v.1.4.0 programme (Rambaut 2016) and edited with Adobe illustrator CS6.

## ﻿Results

### ﻿Phylogenetic analyses

Phylogenetic analyses were constructed with combined 6-locus sequences data representing 73 taxa of Ophiocordycipitaceae. The concatenated LSU-ITS-SSU-*tef1-α*-*rpb1*-*rpb2* data matrix was subjected to Maximum Likelihood (ML) and Bayesian Inference (BI) analyses. Trees were rooted to *Cordycepsmilitaris* in Cordycipitaceae. The alignment contains 4831 characters, including gaps (834 bp for LSU, 506 bp for ITS, 1022 bp for SSU, 918 bp for *tef1-α*, 665 bp for *rpb1* and 886 bp for *rpb2*). The likelihood of the best scoring ML tree was -50301.608. The respective best-fit models determined by ModelFinder on IQ-TREE were GTR+F+I+G4 for LSU, TIM3+F+I+G4 for ITS, K2P+I+G4 for SSU, TIM2+F+I+G4 for TEF1-α, TN+F+I+G4 for RPB1 and RPB2.

**Figure 1.**
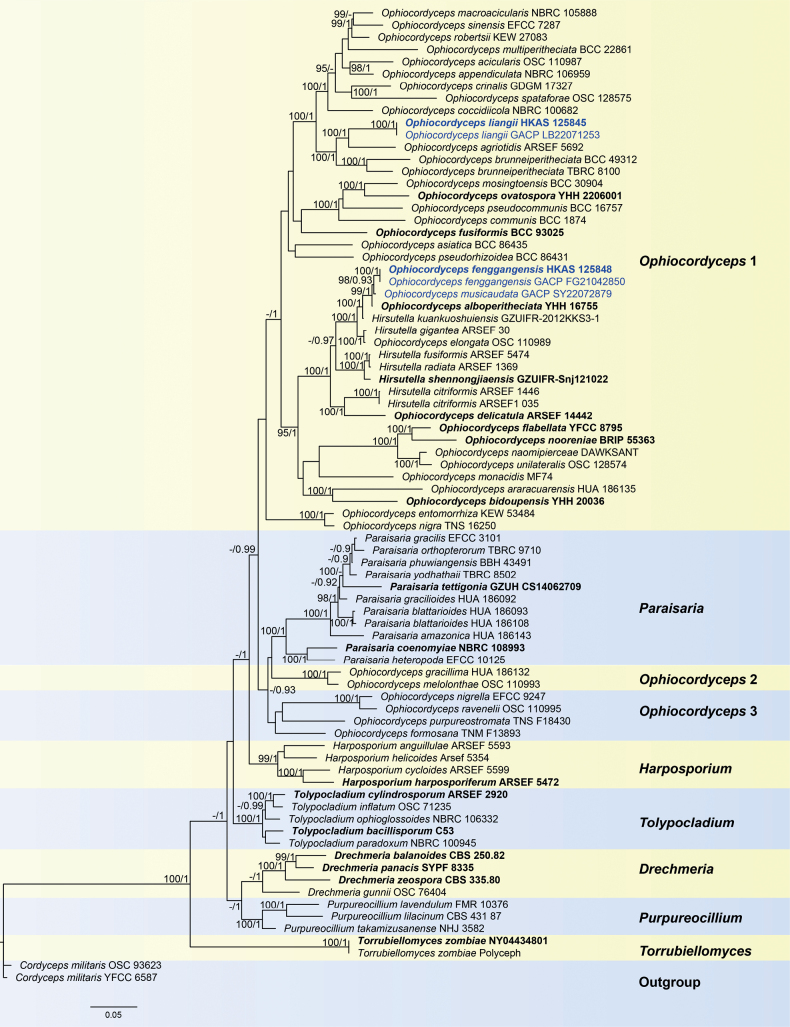
Phylogram generated from Maximum Likelihood analysis, based on combined LSU, ITS, SSU, *tef1-α*, *rpb1* and *rpb2* sequence data. ML bootstrap values equal to or greater than 95% and the PP equal to or greater than 0.90 are given above each node. The newly-generated sequences are indicated in blue. Type strain, type specimens or neotype are denoted in black bold.

In the phylogenetic analyses (Fig. 1), seven genera of Ophiocordycipitaceae are included and their names were labelled on the right side of the tree. The phylogenetic results indicated that the two new species *Ophiocordycepsfenggangensis*, *O.liangii* and one new combination *O.musicaudata* are distinct from other known species. *Ophiocordycepsfenggangensis* and *O.musicaudata* form a monophyletic clade close to *O.alboperitheciata* and *Hirsutellakuankuoshuiensis* with strong support (100% ML / 1.00 PP, Fig. 1). *Ophiocordycepsliangii* (HKAS 102546) sister to *O.agriotidis* with strong support (100% ML/1.00 PP, Fig. 1).

### ﻿Taxonomy

#### ﻿ Ophiocordyceps fenggangensis

Taxon classificationFungiHypocrealesOphiocordycipitaceae

X. C. Peng & T. C. Wen sp. nov.

D8102219-1BAD-5323-9C91-AE122DEAAB93

Index Fungorum: IF901112

Facesoffungi Number: FoF14887

Fig. 2

##### Etymology.

Named after the location where the type specimen was found, Fenggang County, Guizhou Province, China.

**Figure 2.**
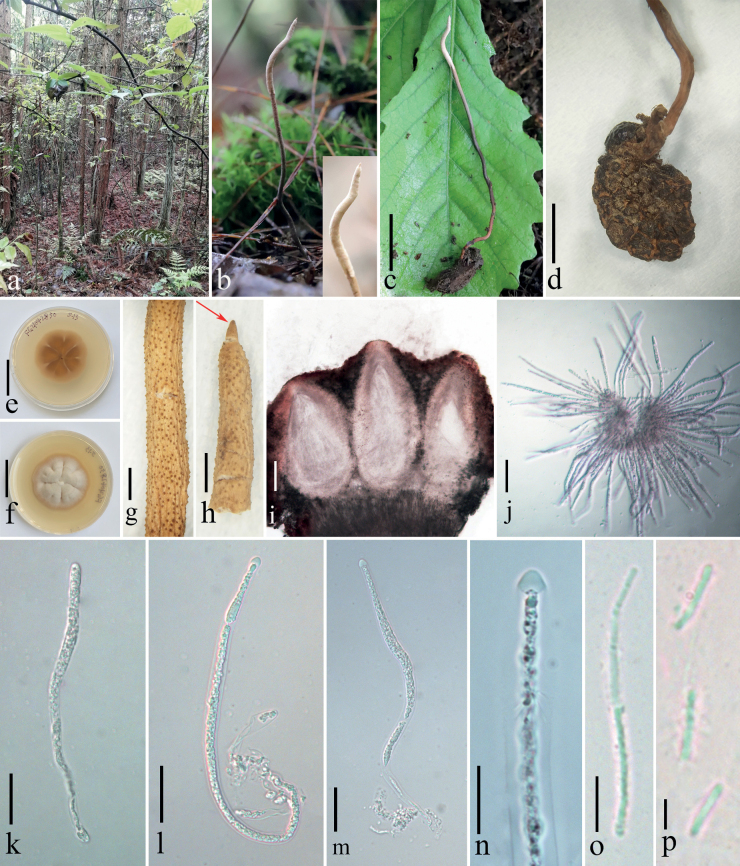
*Ophiocordycepsfenggangensis* (holotype HKAS 125848) **a** habitat **b** host imbedded into the soil with the stroma emerging from the ground **c** stroma arising from the larva of Lepidoptera**d** host **e, f** reverse and front view of the culture on PDA **g** part of fertile head **h** part of fertile head with sterile tip (arrow indicate) **i** perithecia **j–m** asci **n** ascus cap **o** part of ascospores **p** secondary ascospores. Scale bars: 2 cm (**c, e, f**); 5 mm (**d**); 1 mm (**g, h**); 100 µm (**i, j**); 25 µm (**k–m**); 10 µm (**n**); 5 µm (**o**); 2 µm (**p**).

##### Diagnosis.

Parasitic on a larva of Lepidoptera. Stroma arising from the junction between head and thorax of lepidopteran larva, with a sterile tip. Perithecia immersed, grey-white.

##### Sexual morph.

***Stroma*** solitary, unbranched, brown to grey-white, 102 × 1–1.5 mm. ***Fertile part*** up to 24 × 1.5 mm, cylindrical, attenuated toward the apex, grey-white when fresh, yellowish when dry, surface spinous due to the protruding ostioles, with a sterile tip (ca. 0.5 mm in length). ***Stipe*** cylindrical, brown to black, fibrous, 77.5 × 1–1.2 mm. ***Perithecia*** 306–496 × 134–223 μm (x̄= 388.4 × 175.9 µm, σ = 57.35 × 31.05, n = 15), immersed, ovoid to oblong-ovate. ***Asci*** 91–176 × 2–8 μm (x̄= 136.5 × 5.3 µm, σ = 38.22 × 2.63, n = 20), cylindrical, hyaline, with thickened apex. ***Apical cap*** 2.5–5.0 × 3.5–5.6 μm (x̄= 3.6 × 4.7 µm, σ = 0.78 × 0.48, n = 20), hyaline, hemispherical. ***Ascospores*** 0.3–0.7 µm (x̄= 0.4 µm, σ = 38.22 × 2.63, n = 20) wide, filiform, hyaline, easily breaking into part-spores. ***Secondary ascospores*** 2.8–6.0 × 0.3–0.7 μm (x̄= 4.0 × 0.4 µm, σ = 0.89 × 0.08, n = 20), cylindrical, smooth-walled. **Asexual morph**: undetermined.

##### Culture characteristics.

Colonies on PDA, attaining a diameter of 28–32 mm within 39 d at 20 °C, dense, leathery, cream white, convex, undulate margin, reverse brown, radial striation, no sporulation observed.

##### Material examined.

China, Guizhou Province, Fenggang County, Yongan Town (28°05′30.83″N, 107°31′53.38″E, alt. 1149 m), on dead larva of Lepidoptara, 28 April 2021, Xing-Can Peng, FG21042850 (HKAS 125848 holotype, GACP FG21042850 ex-type living culture).

##### Notes.

Multigene phylogenetic analysis showed that *Ophiocordycepsfenggangensis* forms a sister clade to *O.musicaudata* with a high support value (98% ML / 0.93 PP) and grouped with *O.alboperitheciata* and *Hirsutellakuankuoshuiensis* (Fig. 1). *Ophiocordycepsfenggangensis*GACP FG21042850 and *O.musicaudata*GACP SY22072879 have 8 bp differences of nucleotides (0 bp in LSU, 3 bp in *tef1-α* and 5 bp in *rpb1*). Morphologically, *Ophiocordycepsfenggangensis* is distinguished from *O.musicaudata* in having a solitary unbranched shorter stroma, longer perithecia, smaller asci, narrower ascospores and disarticulating ascospores. *Ophiocordycepsalboperitheciata* is distinct from *O.fenggangensis* by its superficial, white to nearly light brown fertile part and ovoid perithecia (Fan et al. 2021), whereas our new species has grey-white to yellowish fertile part and immersed, ovoid to oblong-ovate perithecia. Additionally, the stroma of *O.fenggangensis* is longer than that of *O.alboperitheciata*. Perithecia and asci of *O.fenggangensis* are smaller than those of *O.alboperitheciata*. *Hirsutellakuankuoshuiensis* was described only from its asexual morph which is characterised by clavate, narrow fusiform or botuliform conidia; and subulate or slender columnar phialides tapering gradually to a long narrow neck (Qu et al. 2021). BLAST search result showed that the ex-type strain (GACP FG21042850) matches *Hirsutellakuankuoshuiensis* GZUIFR-2012KKS3-1; however, they are different in 59 bp (including 1 gap) and 8 bp (including 1 gap) within ITS and *rpb1* sequences, respectively. The detailed comparisons of the morphologies between these four aforementioned species are shown in Table 2. Based on the morphological differences, we introduce this fungus as a new species of *Ophiocordyceps*.

**Table 2.** Synopsis of *Ophiocordyceps* species discussed in the paper.

Species	Host	Stromata (mm)	Perithecia(μm)	Asci (μm)	Ascospores (μm)	Reference
***Ophiocordycepsliangii***	larvae of Lepidoptera	113–188 × 2, paired, cylindrical, unbranched, brown to dark brown	350–548 × 203.5–446, superficial, brown, obovoid	122–271.5 × 3.5–13.5, filiform, 8-spored, with thickened apices	67.5–270.5 × 1.5–4.0, filiform to spindle, non-disarticulating	This study
*Ophiocordycepsagriotidis*	larvae of Elateridae, Coleoptera	70 × 1, solitary, cylindrical, black brown to black	400–480 × 225–300, superficial to pseu-immersed, ovoid	235–300 × 12, cylindrical, with an oblate apical cap	115–150 × 4.2–45, cylindricial, multi-septate, non-disarticulating	Liang (2007)
*Ophiocordycepsbrunneiperitheciata*	Lepidopteran larvae	4–8 × 0.5–1, paired to multiple, simple, wiry to pliant or fibrous	350–400 × 180–200, superficial, brown to dark brown, ovoid	125–175 × 6–8, cylindrical, 8-spored, with thickened apices	110–160 × 3–4, filiform, multi-septate, non-disarticulating	Luangsa-Ard et al. (2018)
***Ophiocordycepsfenggangensis***	larvae of Lepidoptera	102 × 1–1.5, solitary, cylindrical, brown to off-white	306–496 × 134–223, immersed, off-white to yellowish, ovoid to oblong-ovate.	91–176 × 2–8, cylindrical, apex thickened	0.3–0.7 wide, filiform, hyaline, disarticulating, secondary ascospores 2.8–6.0 × 0.3–0.7, cylindrical	This study
*Ophiocordycepsalboperitheciata*	larva of Noctuidae, Lepidoptera	69–71 × 0.6–1.2, paired, cylindrical, unbranched, with a sterile tip, light brown to dark brown	410–550 × 230–320, superficial, white to pale brown, nearly ovoid	144–246 × 3.5–4.7, cylindrical, 8-spored, with a hemispheric apical cap	0.5–0.6 wide, multi-septate, non-disarticulating	Fan et al. (2021)
***Ophiocordycepsmusicaudata***	larvae of Lasiocampidae, Lepidoptera	130–140 × 1–2, solitary or numerous, simple or branched, cylindrical, brown to yellowish	260–492 × 144–314, immersed, yellowish, flask-shaped.	123–264 × 5–13, filiform, cylindrical, 8-spored, usually without thickened apices	114–298 × 1.5–4.0, cylindrical, irregular multi-septate, non-disarticulating	This study
*Ophiocordycepsmusicaudata*	larvae of Lasiocampidae, Lepidoptera	up to 165 in length, twin, unbranched, light brown to white	420 × 210, immersed, pseudo-oval	230 × 7.6, cylindrical, with short cylindrical apices	filiform, multi-septate	Liang et al. (1996)
*Ophiocordycepslarvarum*	larva of Lepidoptera	90 × 3.5, solitary, cylindrical, cinnamon light brown	340–380 × 160–200, pseudo-embedding, oblong	180–200 × 8.5, with a hemispheric ascus cap	4-9 × 2-2.5, columnar, septate	Liang et al. (2007)
*Cordycepsochraceostromata*	larva of Lepidoptera	up to 60 in length, single or paired, cylindrical, pale ochraceous-reddish to brownish	350 × 200, immersed, ovoid	up to 7 in width, with thickened apices	disarticulating, secondary ascospores 7–10 × 1.5–2, truncated on both sides	Kobayasi and Shimizu (1980)
*Ophiocordycepszhangjiajiensis*	pupa of Lepidoptera	100 × 2, single or paired, cylindrical, not ramified, leathery, brown to snuff-coloured	330–375 × 180–230, pseudo-embedding, ovoid	200 × 10, approximately fusiform, with thickened apices	disarticulating, secondary ascospores 15–23 × 3, cylindrical	Liang et al. (2002)
*Ophiocordycepsdayiensis*	larva of Lepidoptera	140 × 2, single, filiform, unbranched, brownish	430–480 × 210–270, immersed, narrowly ovoid	225–345 × 6–7.5, slender cylindric, with very thin cap of ascus	300 × 1–1.8, filiform, multi-septate, non-disarticulating	Liang et al. (2003)
*Ophiocordycepsemeiensis*	larva of Hepialidae, Lepidoptera	100–160 × 1.5–3, single or paired, branched, brown	320–460 × 220–320, superficial, brown to black, ellipsoid or ovoid	173–213 × 7.5–8, cylindrical, with hemisphacris heads	45–60 × 1–1.5, filiform, multi-septate	Liang et al. (2007)
*Ophiocordycepslaojunshanensis*	Larvae of Hepialidae, Lepidoptera	47–93 × 1–3.9, simple, rarely 2 or 3, apex sterile acuminate, purplish to dark brown	200–300 × 200–350, globoid, arranged loosely in irregular lateral cushions.	165–275 × 11.5–14.5, clavate	130.0–250 × 5–6, filiform, septate	Chen et al. (2011)
*Ophiocordycepspaludosa*	larvae of Lepidoptera	55–130 × 0.5–1.0, slender filiform, greyish-brown	800–855 × 375–410, superficial, greyish-brown to deep brown, flattened-ovoid	480–550 × 8–10, cylindrical	390–490 × 2.0–2.5, filiform, multi-septate, non-disarticulating	Mains (1940)

#### ﻿ Ophiocordyceps liangii

Taxon classificationFungiHypocrealesOphiocordycipitaceae

X. C. Peng & T. C. Wen sp. nov.

E76DDC5F-E42B-5D07-A09F-5E57A5A0687D

Index Fungorum: IF901113

Fig. 3

##### Etymology.

Named in honour of Prof. Zong-Qi Liang, who has made a significant contribution to the studies of Cordyceps sensu lato.

**Figure 3.**
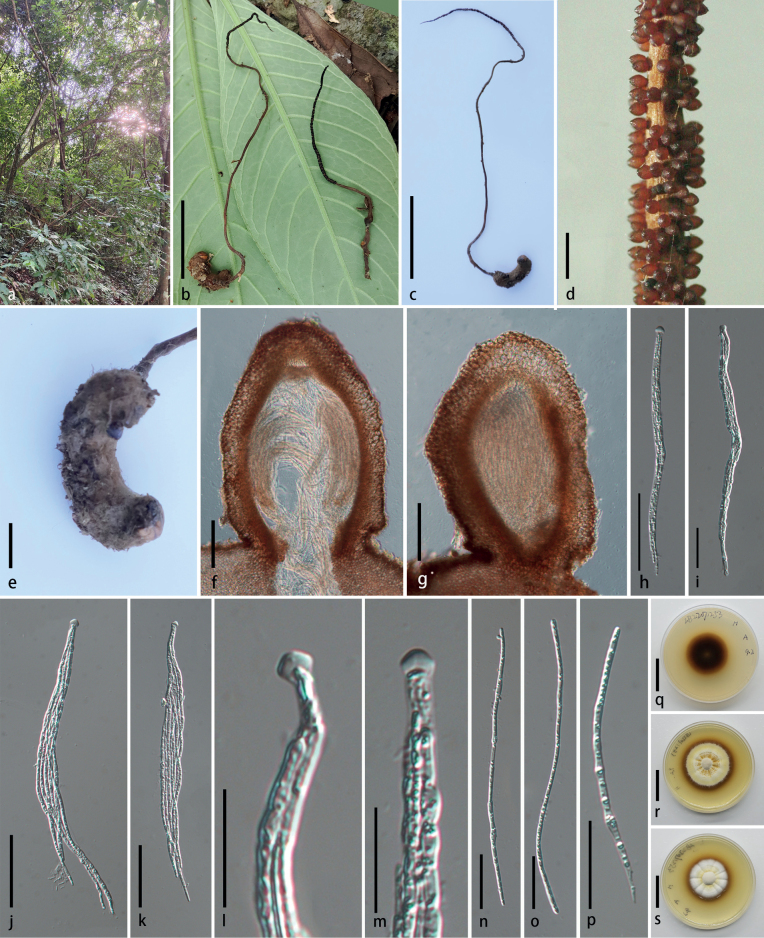
*Ophiocordycepsliangii* (holotype HKAS 125845) **a** habitat **b, c** stromata arising from host **d** superficial perithecia **e** host **f, g** section of perithecia **h–k** asci **h–i** immature **j, k** mature **l, m** ascus cap **n–p** ascospores **q–s** reverse and front view of culture on PDA. Scale bars: 4 cm (**b, c**), 1 mm (**d**), 5 mm (**e**), 100 µm (**f, g**), 50 µm (**h–k**), 20 µm (**l, m**), 30 µm (**n–p**), 2 cm (**q–s**).

##### Diagnosis.

Parasitic on lepidoptaran larva. Stroma arising from the back and tail of host, no sterile tip. Perithecia superficial, dark brown.

##### Sexual morph.

***Stroma*** paired, flexuous, fibrous, filiform, tapering gradually towards the apex, unbranched, brown to dark brown, 11.3–18.8 × 0.2 cm. ***Fertile part*** cylindrical, dark brown, 5.4–6.1 × 0.2 cm. ***Stipe*** flexuous, brown, 5.1–13.5 × 0.1–0.2 cm. ***Perithecia*** 350–548 × 203.5–446 μm (x̄= 430.5 × 296 µm, σ = 56.45 × 60.83, n = 25), superficial, brown, obovoid. ***Asci*** 122–271.5 × 3.5–13.5 μm (x̄= 204.8 × 8.0 µm, σ = 38.22 × 2.63, n = 40), filiform, 8-spored, cylindrical, with thickened apices. ***Apical cap*** 1.7–4.5 × 4.0–6.6 μm (x̄= 3.2 × 5.4 µm, σ = 0.56 × 0.59, n = 40), hyaline, conspicuous. ***Ascospores*** 67.5–270.5 × 1.5–4.0 µm (x̄= 151.3 × 2.6 µm, σ = 36.31 × 0.61, n = 55), fusiform to filiform, aseptate, guttulate, non-disarticulating. **Asexual morph**: undetermined.

##### Culture characters.

Colonies on PDA, attaining a diameter of 21–27 mm within 25 d at 25 °C, dense, leathery, pale yellow at centre, white at periphery, radially striate, with brown or translucent droplets, reverse black brown, producing brown pigment. Sporulation not observed.

##### Material examined.

China, Guizhou Province, Libo County, Xiaoqikong Scenic Area (25°15′15.68″N, 107°43′43.98″E, alt. 458 m), on dead larva of Lepidoptara, on leaf litter, 12 July 2022, Xing-Can Peng, LB22071253 (HKAS 125845 holotype, GACP LB22071253 ex-type culture).

##### Notes.

Phylogenetic analyses revealed that *Ophiocordycepsliangii* is closely related to *O.agriotidis* and *O.brunneiperitheciata* with high support (100% ML/1.00 PP, Fig. 1). *Ophiocordycepsliangii* differs from *O.brunneiperitheciata* and *O.agriotidis* in having longer stroma, larger perithecia and asci (see Table 2). The comparison of the nucleotide sequences between *O.liangii* (GACP LB22071253) and *O.brunneiperitheciata* (TBRC 8100) showed 23 bp (including 3 gaps) differences in LSU, 102 bp in *tef1-α* and 88 bp in *rpb2* sequences. *Ophiocordycepsliangii* differs from *O.agriotidis* ARSEF 5692 by 3 bp in SSU, 70 bp (including 20 gaps) in ITS, 20 bp (including 1 gap) in LSU, 106 bp in *tef1-α* and 79 bp in *rpb2*. Henceforth, we describe this taxon as a new species in *Ophiocordyceps*.

#### ﻿ Ophiocordyceps musicaudata

Taxon classificationFungiHypocrealesOphiocordycipitaceae

(Z. Q. Liang & A. Y. Liu) X. C. Peng & T. C. Wen comb. nov.

0A68C1B2-5E7B-50D4-B8EB-BA40392F784B

Index Fungorum: IF901114

Facesoffungi Number: FoF14889

Fig. 4

Cordyceps musicaudata Z. Q. Liang & A. Y. Liu. Basionym.

##### Diagnosis.

Parasitic on larvae of insect (Lasiocampidae, Lepidoptera). Stroma arising from body of the host, no sterile tip. Perithecia immersed, yellowish.

**Figure 4.**
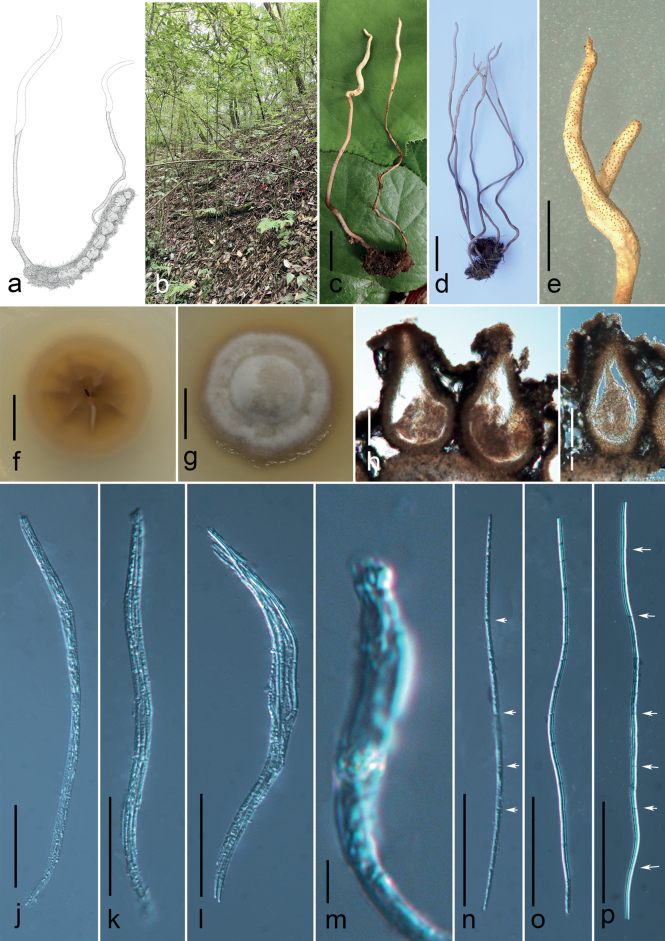
*Ophiocordycepsmusicaudata* (HKAS 131911) **a** redrawn of Liang (2007)**b** habitat **c, d** stromata arising from host **e** fertile parts **f, g** reverse and front view of culture on PDA **h, i** perithecia **j–l** asci **m** ascus cap **n–p** ascospores, the arrows in the **n, p** indicating septate. Scale bars: 2 cm (**c, d**); 5 mm (**e**); 1 cm (**f, g**); 100 µm (**h, i**); 50 µm (**j–l, n–p**); 5 um (**m**).

##### Sexual morph.

***Stroma*** solitary, paired to multiple, simple or branched, flexuous, cylindrical with acute or round ends, 13–14 × 0.1–0.2 cm. ***Fertile part*** cylindrical, yellowish, 2–4.3 × 0.1–0.2 cm. ***Stipe*** flexuous, brown, 10–12 × 0.1 cm. ***Peridium*** 15–49 µm (x̄= 33 µm, σ = 8.41, n = 40) wide, composed of brown cells of *textura angularis*. ***Perithecia*** 260–492 × 144–314 μm (x̄= 378 × 221 µm, σ = 37.29 × 1.57, n = 25), immersed, flask-shaped. ***Asci*** 123–264 × 5–13 μm (x̄= 191 × 8.1 µm, σ = 58.76 × 48.94, n = 80), cylindrical, 8-spored, with inconspicuous thickened cap. ***Ascospores*** 114–298 × 1.5–4.0 µm (x̄= 198 × 2.3 µm, σ = 46.03 × 0.48, n = 45), filiform, irregular multi-septate, non-disarticulating. **Asexual morph**: undetermined.

##### Culture characteristics.

Colonies on PDA, attaining a diameter of 21–27 mm within 43 d at 25 °C, dense, velvety, off-white, wrinkled bulge, reverse brown. No sporulation observed.

##### Epitype designated here.

China, Guizhou Province, Suiyang County, Kuankuoshui National Nature Reserve (28°13′N, 107°09′E, alt. 1470–1507 m), on dead larva of Lepidoptera sp. buried in soil, 28 July 2022, Xing-Can Peng, SY22072880 (HKAS 131911 epitype); Ting-Chi Wen, SY22072879 (HKAS 131912, GACP SY22072879, live culture).

##### Notes.

Liang et al. (1996) published a new species, *Cordycepsmusicaudata* solely based on morphological observation. The type specimen (CGAC89-62301) was found on the insect (Lasiocampidae, Lepidoptera) in the Kuankuoshui National Nature Reserve, Guizhou Province, China. It is regrettable that the type specimen has been destroyed, thus its DNA and morphological observations could not be obtained. Liang et al. (1996) stated that the type specimen has characteristics of paired rat-tailed stromata, white to pale brown fertile part, brown stipe, immersed perithecia, cylindrical asci with thickened apices and filiform, multi-septate ascospores. In this study, we collected two fresh specimens from the same location to the type specimen. The fresh specimen shares similar morphology with the type specimen of *C.musicaudata* in the lepidopteran hosts, stipitate rat-tailed stromata with yellowish fertile part, immersed perithecia and filiform, multi-septate, intact ascospores. Phylogenetic analysis indicated that *C.musicaudata* has close affinity with *O.alboperitheciata* and *O.fenggangensis* with adequate support (99% ML / 1 PP, Fig. 1). The differences between *C.musicaudata* and *O.fenggangensis* have been mentioned in the notes of *O.fenggangensis*. The difference between *C.musicaudata* and *O.alboperitheciata* is the size and the arrangements of the perithecia. *Ophiocordycepsmusicaudata* has smaller and immersed perithecia, whereas *O.alboperitheciata* has larger and superficial perithecia. The detailed comparisons of morphologies between our specimen and related species including species without molecular data are shown in Table 2 (*Cordycepsochraceostromata*, *Ophiocordycepsalboperitheciata*, *O.dayiensis*, *O.emeiensis*, *O.fenggangensis*, *O.laojunshanensis*, *O.larvarum*, *O.zhangjiajiensis* and *O.paludosa*). Our specimen morphologically more matches *Cordycepsmusicaudata* rather than other *Ophiocordyceps* species included in the Table 2. Therefore, we determined these specimens as *Cordycepsmusicaudata* and move this species into the genus *Ophiocordyceps*, based on the phylogenetic affiliation of this new collection.

## ﻿Discussion

It has been observed that there are eight genera in Ophiocordycipitaceae that possess versatile lifestyles (Crous et al. 2020; Araújo et al. 2022; Xiao et al. 2023). *Drechmeria* typically live as endoparasites inside nematodes and lepidopteran larvae (Yu et al. 2018). *Hantamomyces* is a monotypic genus that was established by Crous et al. (2020), based on *H.aloidendri* found on the leaves of *Aloidendrondichotomum*. Most species of *Harposporium* parasitise free-living nematodes and rotifers; however, the taxonomic status of some species in this genus is still difficult to determine (Crous et al. 2023). *Paraisaria* accommodate 18 species that were established due to their distinctive features, such as the fleshy, robust solitary stroma, globose to ovoid fertile head and brighter colour. These characteristics are different from other Ophiocordycipitaceae species (Mongkolsamrit et al. 2019). *Purpureocillium* contains six species that are entomopathogenic fungi> or pathogenic to humans (Luangsa-Ard et al. 2011). Species of *Tolypocladium* infect hosts crossing animals, plants and fungi>, showing highly diverse lifestyles (Yu et al. 2021). *Torrubiellomyces* is a genus with only one species that is a mycoparasite. The species has superficial perithecia that grow directly on the host’s surface (Araújo et al. 2022). Genera of Ophiocordycipitaceae are monophyletic with the exception of *Ophiocordyceps* which has been split into three clades due to erection of *Paraisaria* (Mongkolsamrit et al. 2019; Wei et al. 2021b; Wei et al. 2022). So far, there are 419 species in the *Ophiocordyceps*, including 98 unclarified *Hirsutella* species (until 28 Aug 2023). Amongst them, molecular data are not available for 194 species. In this study, 75 taxa representing 70 species of *Ophiocordyceps* are sampled and used for phylogenetic analysis. The topologies of the main clades are similar to previous studies (Wei et al. 2022; Xiao et al. 2023). Insertion of *Paraisaria* causes paraphyly of *Ophiocordyceps*; *Drechmeria* and *Purpureocillium* form a clade sister and *Harposporium* forms a clade sister with *Ophiocordyceps* s. s. and *Paraisaria* (Xiao et al. 2023). The sexual morphs of *Ophiocordyceps* species phenotypically share a darkly or brightly coloured, fibrous stromata often with aperithecial apices or lateral pads. Perithecia are superficial to completely immersed, ordinal or oblique in arrangement. Asci are cylindrical to filiform with thickened apex. Ascospores are cylindrical, multi-septate, disarticulating into secondary spores or not (Sung et al. 2007). However, they can be distinguished according to their associated host, arrangement of perithecia, size, shape, colour of fertile part and morphologies of ascospores and part-spores. Notably, combined molecular phylogenetic analysis provides further evidence of their interspecific relationship.

Most of the fungal species published before the 1990s relied on classical morphology to determine the taxonomic status. It is difficult to gain access to their molecular data and morphological illustration and other related information as well as their type specimens. These issues emphasise the importance of collecting fresh specimens and clarifying them with modern approaches. Such work has been conducted by Sung et al. (2007) who systematically classified *Cordyceps* and clavicipitaceous fungi> through molecular phylogenetic analysis and revised most of the species of *Cordyceps* s. l. Henceforth, an increasing number of new species were described and the natural classification of hypocrealean entomopathogens were gradually elucidated, based on more sufficient taxa sampling (Ban et al. 2009; Evans et al. 2011; Sanjuan et al. 2015; Simmons et al. 2015; Spatafora et al. 2015; Araújo et al. 2018; Khonsanit et al. 2019; Araújo et al. 2020; Mongkolsamrit et al. 2021; Yang et al. 2021; Araújo et al. 2022; Mongkolsamrit et al. 2022; Tang et al. 2022; Mongkolsamrit et al. 2023; Tang et al. 2023a, b). However, *Cordycepsmusicaudata* has not been revised because there are no specimens available for study of its morphological and molecular data. We have conducted a study wherein fresh specimens were collected from the same location as the type of *Cordycepsmusicaudata*. Our observations reveal that there are certain similarities between the fresh specimen and some species mentioned in Table 2, both at a macroscopic and microscopic level. However, we also observed noticeable differences between them. For example, the perithecia of *Ophiocordycepslarvarum* and *O.zhangjiajiensis* are pseudo-immersed; *O.emeiensis* and *O.paludosa* have superficial perithecia; the ascospores of *O.dayiensis* are slender; the stromata of *O.laojunshanensis* are short and the perithecia are globoid; and the ascospores of *Cordycepsochraceostromata* disarticulate into secondary spores (see Table 2). Based on molecular analysis and updated morphological illustration, we identified the specimen as *Cordycepsmusicaudata* and synonymised it as *O.musicaudata*. Moreover, two new species, *O.fenggangensis* and *O.liangii* are described from their sexual morphs and phylogenetic results support their novelty.

## Supplementary Material

XML Treatment for Ophiocordyceps fenggangensis

XML Treatment for Ophiocordyceps liangii

XML Treatment for Ophiocordyceps musicaudata

## ﻿Additional information

### Author ORCIDs

Xing-Can Peng https://orcid.org/0000-0002-7271-7639

Ting-Chi Wen https://orcid.org/0000-0003-1744-5869

De-Ping Wei https://orcid.org/0000-0002-3740-0142

Yi Wang https://orcid.org/0009-0006-5412-7893

Xian Zhang https://orcid.org/0009-0008-0919-4303

Khanobporn Tangtrakulwanich https://orcid.org/0009-0002-7081-618X

Jian-Dong Liang https://orcid.org/0000-0002-3939-3900

### Data availability

All of the data that support the findings of this study are available in the main text.
